# Building a Transnational Biosurveillance Network Using Semantic Web Technologies: Requirements, Design, and Preliminary Evaluation

**DOI:** 10.2196/jmir.2043

**Published:** 2012-05-29

**Authors:** Douglas Teodoro, Emilie Pasche, Julien Gobeill, Stéphane Emonet, Patrick Ruch, Christian Lovis

**Affiliations:** ^1^University Hospitals of GenevaGenevaSwitzerland; ^2^University of GenevaGenevaSwitzerland; ^3^University of Applied Sciences Western SwitzerlandGenevaSwitzerland

**Keywords:** Antimicrobial drug resistance, heterogeneous databases, online information services, surveillance

## Abstract

**Background:**

Antimicrobial resistance has reached globally alarming levels and is becoming a major public health threat. Lack of efficacious antimicrobial resistance surveillance systems was identified as one of the causes of increasing resistance, due to the lag time between new resistances and alerts to care providers. Several initiatives to track drug resistance evolution have been developed. However, no effective real-time and source-independent antimicrobial resistance monitoring system is available publicly.

**Objective:**

To design and implement an architecture that can provide real-time and source-independent antimicrobial resistance monitoring to support transnational resistance surveillance. In particular, we investigated the use of a Semantic Web-based model to foster integration and interoperability of interinstitutional and cross-border microbiology laboratory databases.

**Methods:**

Following the agile software development methodology, we derived the main requirements needed for effective antimicrobial resistance monitoring, from which we proposed a decentralized monitoring architecture based on the Semantic Web stack. The architecture uses an ontology-driven approach to promote the integration of a network of sentinel hospitals or laboratories. Local databases are wrapped into semantic data repositories that automatically expose local computing-formalized laboratory information in the Web. A central source mediator, based on local reasoning, coordinates the access to the semantic end points. On the user side, a user-friendly Web interface provides access and graphical visualization to the integrated views.

**Results:**

We designed and implemented the online Antimicrobial Resistance Trend Monitoring System (ARTEMIS) in a pilot network of seven European health care institutions sharing 70+ million triples of information about drug resistance and consumption. Evaluation of the computing performance of the mediator demonstrated that, on average, query response time was a few seconds (mean 4.3, SD 0.1×10^2^ seconds). Clinical pertinence assessment showed that resistance trends automatically calculated by ARTEMIS had a strong positive correlation with the European Antimicrobial Resistance Surveillance Network (EARS-Net) (ρ = .86, *P *< .001) and the Sentinel Surveillance of Antibiotic Resistance in Switzerland (SEARCH) (ρ = .84, *P *< .001) systems. Furthermore, mean resistance rates extracted by ARTEMIS were not significantly different from those of either EARS-Net (∆ = ±0.130; 95% confidence interval –0 to 0.030; *P *< .001) or SEARCH (∆ = ±0.042; 95% confidence interval –0.004 to 0.028; *P *= .004).

**Conclusions:**

We introduce a distributed monitoring architecture that can be used to build transnational antimicrobial resistance surveillance networks. Results indicated that the Semantic Web-based approach provided an efficient and reliable solution for development of eHealth architectures that enable online antimicrobial resistance monitoring from heterogeneous data sources. In future, we expect that more health care institutions can join the ARTEMIS network so that it can provide a large European and wider biosurveillance network that can be used to detect emerging bacterial resistance in a multinational context and support public health actions.

## Introduction

Since their discovery, antibiotics have proved powerful for the control of bacterial infections. However, because of multifactorial causes, especially the widespread use of antibiotics in medicine, animal husbandry, and agriculture, pathogens have developed increasing resistance to many effective drugs [1,2]. The problem of antimicrobial resistance has reached an alarming level, and urgent efforts are needed to avoid regressing to the preantibiotic era [3,4].

In addition to well-known drug resistance cases such as *Pneumococcus *species to penicillin [5-7], outbreaks of new resistant pathogens have become ever more common and have caused many deaths worldwide. Aware of the risks that antimicrobial resistance poses to global public health, the World Health Organization (WHO), among other measures, chose combating antimicrobial resistance as the theme of World Health Day 2011. Lack of effective monitoring systems was identified as an underlying cause of resistance increase, and its improvement is one of the policies the WHO adopted to tackle the problem[8].

Over a decade ago, Monnet et al[9] described and compared the most relevant antimicrobial resistance surveillance systems in Europe. Since then, no new public transnational surveillance initiatives have been developed[10]. Consequently, most projects in use are based either on reporting and manual data acquisition or on outdated information technologies, especially concerning data integration and semantics. Furthermore, no cross-country surveillance system that provides online, direct, and real-time access to antimicrobial resistance information is available. All the systems implemented so far are dependent on delayed data warehouses, usually compiled yearly, which, among other weaknesses, fail to capture antimicrobial resistance outbreaks[10,11]. Finally, these systems do not provide easy ways to export data. Participating institutes have to comply with the surveillance system standards, a labor intensive task, especially for newcomer institutions or newly discovered resistance pathogens [11].

The primary aim of this study was to develop a framework for transnational antimicrobial resistance monitoring, featuring real-time access to laboratory information and being generic with respect to data sources, in order to support multinational resistance surveillance. The secondary aim of the study was to investigate the use of Semantic Web-based architecture in the integration and interoperability of interinstitutional and cross-border databases to support such a framework. To fulfill these aims, we designed the Antimicrobial Resistance Trend Monitoring System (ARTEMIS). ARTEMIS architecture illustrates how Semantic Web technologies can support online monitoring of antimicrobial resistance trends in heterogeneous networks of health care institutions. It demonstrates how semantically interoperable end points can provide on-demand information on resistance evolution. Furthermore, it describes ways to automate the monitoring process through a state-of-the-art clinical data integration system, which provides mechanisms to adapt to existing electronic health records and laboratory information systems. The architecture is validated according to performance and clinical pertinence.

This paper addresses a large audience, from engineers who have Semantic Web techniques in mind to public health authorities, by showing the results of applying Semantic Web technologies to one of the most crucial current public health challenges: building a global surveillance system for antimicrobial resistances. Here we discuss the technical framework of the project, a technical evaluation, and the quality of the system compared with existing surveillance networks.

### Previous European Antimicrobial Resistance Monitoring and Surveillance Initiatives

Several projects have been implemented to provide monitoring and surveillance of antimicrobial resistance evolution in a European context. WHONET was one of the first initiatives to standardize and aggregate results from laboratories in a cross-country environment[12]. Since 1995, the WHO has been developing the WHONET software, in which participating microbiology laboratories present their tests using a specific susceptibility testing terminology defined by the WHO.

The most successful European surveillance project is the European Antimicrobial Resistance Surveillance System[13] developed by the European Centre for Disease Prevention and Control. According to the agency, 900 public health laboratories serving over 1400 hospitals in Europe participate in the network, providing results on a yearly basis. To improve data quality, external control is applied to the susceptibility testing methods used by the participating laboratories. The project has recently evolved into the European Antimicrobial Resistance Surveillance Network (EARS-Net)[14] and will serve as a reference to assess the sampling effectiveness of ARTEMIS.

A few other public initiatives were introduced in parallel. In 1998, the European Society of Biomodulation and Chemotherapy created the European Surveillance of Antibiotic Resistance project[15]. The goal was to establish a representative network of sentinel diagnostic laboratories across Europe to provide antimicrobial resistance monitoring and early detection of new resistant pathogens. In the same year, the US Centers for Disease Control and Prevention launched the International Network for the Study and Prevention of Emerging Antimicrobial Resistance [16] with 79% of participant countries, out of 40, from Europe. The main objective of the project was to serve as an early warning system for emerging resistant pathogens. Finally, in 1999, the Antimicrobial Resistance Information Bank [17] was derived from the WHONET informal network. Results were reported to the WHO, and an additional external audit quality control was performed on the data. All of these projects have been discontinued, and some were characterized more as a survey than as a surveillance system.

In contrast to the previous initiatives, The Surveillance Network is a corporate-funded surveillance project[18]. It started in 1992 in the United States and later enrolled European laboratories as well. The data extraction and aggregation processes are done by Focus Technologies Inc. (Herndon, VA, USA), the company responsible for the project. Unfortunately, despite having probably the biggest antimicrobial resistance database worldwide, this network provides no antimicrobial resistance information free to the public.

### The DebugIT Project

ARTEMIS was developed as part of the Detecting and Eliminating Bacteria Using Information Technology (DebugIT) project, which is funded by the European Union Seventh Framework Programme [19]. DebugIT is a consortium composed of 14 industrial, research, and clinical institutions from nine countries that are collaborating to build a framework for sharing antimicrobial resistance information from clinical information systems in a Europeanwide context. The project aims to reuse existing clinical data for generating new knowledge to be incorporated in decision support and monitoring engines at the point of care and for developing prevention strategies at policy levels.

**Figure 1:**
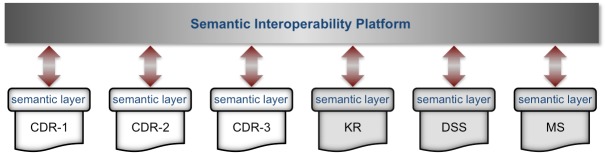
Architecture of the Detecting and Eliminating Bacteria Using Information Technology (DebugIT) framework. Components of the architecture, such as the clinical data repository (CDR), knowledge repository (KR), decision support system (DSS), and monitoring system (MS), are interconnected using the HTTP/SPARQL protocol through the Internet bus. Messages are transferred in the RDF format, and ontologies formalize the data model and content.

The DebugIT architecture (Figure 1) is based on distributed services that exchange information using Semantic Web technologies [20]. The Semantic Web stack provides methods that can contribute to solving technical, syntactic, and semantic differences between disparate data sources [21-24], bringing formal and meaningful representation to data models and sources. First, it presents a standard format to encode information called Resource Description Framework (RDF) [25], which models Web resources in a subject–predicate–object form, a so-called *triple*. This generic model, in contrast to the entity-relationship model used in traditional databases, facilitates the representation of clinical facts to an unconstrained dimension[26]. Second, it has defined the Simple Protocol and RDF Query Language (SPARQL) standard that provides ways to access ubiquitously resources available on the Web[27]. Finally, computer-interpretable ontologies written in the Web Ontology Language [28] bring formal conceptualization to RDF resources, improving the quality of data and fostering interoperability between heterogeneous systems.

## Methods

Experts from the DebugIT project with different backgrounds, including infectiologists, epidemiologists, computer scientists, knowledge engineers, and eHealth service providers, were involved in the design of ARTEMIS. Over the course of 2 years, we held weekly meetings with these experts to discuss the status of the tasks involved in the system development [29]. In the process, we reviewed the existing distributed integration and interoperable eHealth systems and European antimicrobial resistance monitoring programs. Thereafter, we elaborated the requirements and designed the system model.

### Design Requirements

To provide a monitoring system that can be effectively used in the fight against antimicrobial resistance, we derived the following six main requirements based on the published literature and on the expertise of the DebugIT consortium.

#### The System Shall Provide Online Information

All public European supranational monitoring systems provide resistance information in batch mode—that is, data are collected into batches of laboratory tests and processed periodically, usually on a yearly frequency. While online resistance information is useful on a daily basis at local levels, recent infectious pandemic threats have shown how important this information would be at a multinational level for decision makers. Thus, changing this paradigm to online trends is crucial for antimicrobial resistance surveillance, especially for early warning of emerging resistance trends [10,11].

#### The System Shall Provide Aggregated Information From Numerous National Sources

Increasing antibiotic resistance is a worldwide public health concern, and for its effective combat, a successful surveillance system has to offer multinational resistance information[30].

#### The System Shall Not Store Data Centrally

Sharing biomedical data raises several ethical concerns[31]. To comply with international standards on sharing biomedical information, increase the trust of data providers, and encourage collaboration in the surveillance network, central aggregation must be avoided.

#### The System Shall Implement a Formal and Semantic-Aware Data Model

Most of the available systems do not use formalized biomedical data models, nor computable terminologies and ontologies. As a result, the process of extracting resistance information and data analysis in a heterogeneous environment is done manually or semiautomatically. In addition to the overhead work, the lack of formal conceptualization of the raw laboratory data can have a negative influence on the quality of the data.

#### The System Shall be High Performing

To be operatively used by health care professionals, whose working environment is recognized to be very time constrained, eHealth systems must provide a fast response time.

#### The System Shall Provide Reliable Results

Automatic extraction of antimicrobial resistance trends from heterogeneous data sources poses several challenges to accurate data analysis, including concept ambiguity and the common denominator, which can degrade the quality of the examination. However, especially if the system is used by clinicians at the point of care, the accuracy of the results must be equivalent to those obtained by semiautomatic processes, where data cleansing and audit are performed prior to integration and interpretation.

### System Model

**Figure 2:**
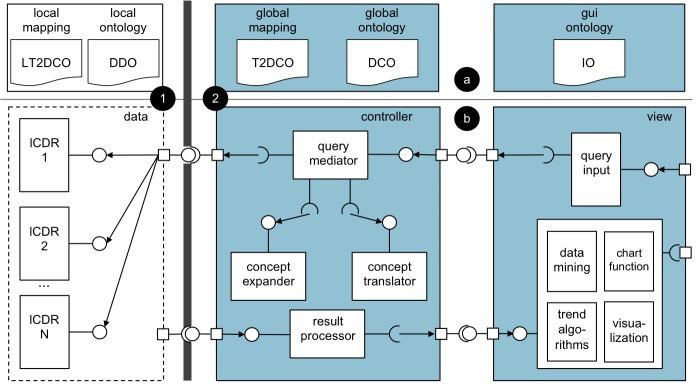
Antimicrobial Resistance Trend Monitoring System (ARTEMIS) architecture. (a) Ontology components. Models: data definition ontology (DDO), DebugIT Core Ontology (DCO), and interface ontology (IO). Mappings: local-terminology-to-DCO (LT2DCO) and global-terminology-to-DCO (T2DCO). (b) Run-time business components. (1) Data layer components are deployed within the demilitarized zone of the health care institution. (2) Controller and view layers contain central services, which are deployed in the Internet. lCDR = local clinical data repository.

To fulfill the ARTEMIS desiderata, we envisaged the system according to the Semantic Web-complying architecture presented in Figure 2. The system’s semantic interoperability schema is based on an ontology-driven data integration approach[32], where multiple semantically flat local data definition ontologies are mapped to a common domain ontology, the DebugIT Core Ontology [33]. Semantic mappings at local and global levels align concepts from the local ontologies with the domain knowledge.

In the architecture’s data model layer, local laboratory databases are connected online to semantic-aware end points, the local clinical data repositories (lCDRs) [34,35]. The lCDRs formalize the local sources and provide a query interface to the controller layer. The semantic mediator, implemented at the controller layer, represents antimicrobial resistance clinical questions as query templates for each end point and coordinates the access to the different sites. It performs the query’s data aggregation operations locally to improve query performance and the site’s data integration on the fly to avoid central storage. Finally, in the view layer, query templates with parameters extracted from the domain ontology are used to represent antimicrobial resistance clinical questions. As a proof of concept, three initial query templates were proposed by clinicians to be implemented in the system. (1) What is the evolution of resistance to *:antibiotic *of *:bacteria *cultured from *:sample *extracted from *:gender *patients at *:clinical_setting *during period *:begin_date *- *:end_date*? (2) What is the prevalence of *:antibiotic* *:susceptibility* *:bacteria *in *:sample *extracted from *:gender *patients at *:clinical_setting *during period *:begin_date *- *:end_date*? (3) What is the rate of *:gender *patients that get *:antibiotic *to treat *:bacteria *infection found in *:sample *at *:clinical_setting *during period *:date_begin *- *:date_end*?

A more detailed description of the system model is given in Multimedia Appendix 1.

### Participants

To assess ARTEMIS, we connected a network of seven data providers: National Heart Hospital, Sofia, Bulgaria; Les Hôpitaux Universitaires de Genève, Geneva, Switzerland; Georges Pompidou European Hospital, Paris, France; Internetový Pristup Ke Zdravotním Informacím Pacienta, Prague, Czech Republic; Swedish Intensive Care Registry, Sweden; Athens Chest Hospital “Sotiria”, Athens, Greece; and Universitätsklinikum Freiburg, Freiburg, Germany. Table 1 summarizes antimicrobial resistance-related data shared by these institutions.

We obtained permission to use de-identified data from the ethics committees of the respective participant hospitals. Privacy-sensitive information accessible through the local end points was pseudoanonymized to conform to the European legal and ethical patient data-sharing framework[36]. Data values such as *date of birth *were truncated to the year, and concepts such as *episode of care *(or *encounter*) and *patient identifiers *were encrypted. Furthermore, query templates are pathogen and population centric—that is, the information collected concerns the resistance and treatment of a pathogen population for a given antibiotic in a set of microbiology results. It is therefore not related to a specific patient.

**Table 1:** Data used in the Antimicrobial Resistance Trend Monitoring System (ARTEMIS).

Data group	Data item	ACH^a^	HEGP^b^	HUG^c^	IZIP^d^	NHH^e^	SIR^f^	UKLFR^g^
Demographics	Age	×^h^	×	×	×	×	×	−^i^
	Sex	×	×	×	×	×	×	−
Location	Department	−	×	−	−	−	−	×
Laboratory	Bacteria	×	×	×	−	×	×	×
	Antibiotic	×	×	×	−	×	×	×
	Specimen	×	×	×	−	×	×	×
	S.I.R.^j^	×	×	×	−	×	×	×
Medication	Drug	×	×	×	×	×	−	−
Triples (×10^6^)		0.05	25.20	19.87	2.79	0.02	3.81	19.10

^a ^Athens Chest Hospital “Sotiria.”

^b ^Georges Pompidou European Hospital.

^c ^Les Hôpitaux Universitaires de Genève.

^d ^Internetový Pristup Ke Zdravotním Informacím Pacienta.

^e ^National Heart Hospital.

^f ^Swedish Intensive Care Registry.

^g ^Universitätsklinikum Freiburg.

^h ^Concept available in the local clinical data repository.

^i ^Concept not available in the local clinical data repository.

^j ^Breakpoint values: susceptible (S), intermediate (I), and resistant (R).

### Outcome Measures

The implementation of the functional features defined in the first four design requirements is described at the technical component level using design pattern [37-39] examples. In contrast, for the last two requirements, which can be quantitatively measured, results are presented using efficiency and effectiveness metrics.

### Methods for Data Acquisition and Analysis

We measured efficiency using the mediator’s query retrieval time for the three aforementioned query templates. Combinations of pathogens, antibiotics, and sample types were applied to vary the queries and thus avoid database caching effects. Results of the local aggregation mode applied in the query mediator were compared with a central aggregation strategy (baseline).

To assess effectiveness, resistance trends extracted using query template 1 were compared with data from two publicly available surveillance systems: EARS-Net and the Sentinel Surveillance of Antibiotic Resistance in Switzerland (SEARCH). We extracted yearly resistance trends for seven key pathogenic bacteria—*Enterococcus faecalis*, *Enterococcus faecium*, *Escherichia coli*, *Klebsiella pneumoniae*, *Pseudomonas aeruginosa*, *Staphylococcus aureus*, and *Streptococcus pneumoniae*—based on their presence in the three systems. Antibiotics were selected if they were present on both ARTEMIS and the reference system. Resistance rates of the last 4 years (2006 to 2009) available in EARS-Net were used, whereas all years (2008 to 2010) available in SEARCH were taken into account. ARTEMIS data sources that did not contain either more than 1 million triples or data elements to answer the queries were excluded from the analysis, resulting in four sites: Georges Pompidou European Hospital, Les Hôpitaux Universitaires de Genève, Swedish Intensive Care Registry, and Universitätsklinikum Freiburg.

We compared results from Georges Pompidou European Hospital, Swedish Intensive Care Registry, and Universitätsklinikum Freiburg with the resistance rates of their respective EARS-Net countries—France, Sweden, and Germany— and results from Les Hôpitaux Universitaires de Genève with SEARCH. We report correlation and equivalence results using the Spearman rank correlation and the two one-sided convolution[40,41] tests, respectively (see Multimedia Appendix 2).

## Results

**Figure 3:**
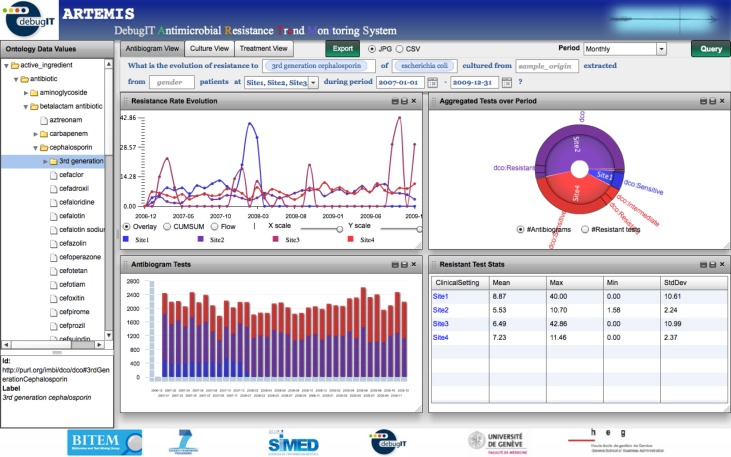
Antimicrobial Resistance Trend Monitoring System (ARTEMIS) interface. The menu on the left displays the interface ontology concepts, which are used to fill in the template parameters. Each of the view tabs represents a different query template. The data visualization interface displays several graphical representations to provide a comprehensive view of the data.

ARTEMIS was implemented and deployed in a pilot network of seven European health care institutions sharing 70+ million triples of antimicrobial resistance information. As Figure 3 shows, near real-time resistance trends can be extracted from the distributed network using the system’s Web interface. The tool can be accessed at http://babar.unige.ch:8080/artemis.

### Model Components

In this section, we present design patterns describing the main functional features of the online distributed monitoring system.

#### Online Information Provider

##### Requirement

The system shall provide online information.

##### Design

In the architecture presented in Figure 2, local semantic-aware end points, realized by RDF stores, are plugged into the laboratory databases. Thus, microbiology tests are accessible as soon as they are available in the production databases. These end points are formalized by local ontologies and exposed to the Web so that data are reachable by other parts of the system. In cases where local laboratory databases communicate in the SPARQL protocol, they can be directly connected to the network.

##### Example

**Figure 4:**
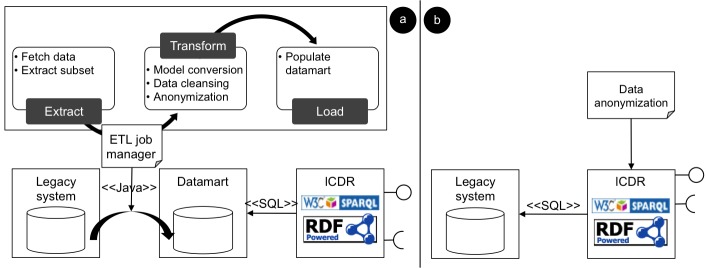
Local clinical data repository (lCDR) deployment and population model. (a) Production data are extracted daily to a local mirror database, which is “sparqlized” by an SQL-to-RDF engine. (b) RDF view is created directly on top of the legacy system. Data are anonymized on the fly.

In ARTEMIS, the technical interoperability with the different data sources is provided by D2R [42] engines complemented by site-specific extract, transform, and load processes (Figure 4, part a), which can exploit autocoding methods [43,44]. Alternatively (Figure 4, part b), for cases where there is an accessible production laboratory database, D2R can be plugged directly into the existing system to transform the local data source into a semantic end point (lCDR).

#### Distributed Storage

##### Requirement

The system shall provide aggregated information from numerous international sources.

##### Design

The technical and semantic heterogeneity within models and concepts from different clinical data sources poses an important barrier for data aggregation and analysis. ARTEMIS architecture relies on a layer of semantically formalized end points, the lCDRs, to solve part of the integration problem. These end points provide a first level of interoperability, modeling the local systems and the data content and providing a common protocol to access data, the SPARQL protocol. The semantic mediator designed in the controller layer builds on top of the lCDR layer and allows the creation of homogeneous aggregated views over the distributed data sources. Thus, the system becomes a grid of semantic-aware sentinels that provide antimicrobial resistance information from heterogeneous supranational data sources.

##### Example

In ARTEMIS, the lCDRs are provided by RDF-like stores to create the first semantic layer on top of the local databases. The data definition ontologies formalize the local end points and expose linkable data on the Web. The Jena Framework is used for querying the remote lCDRs and for reasoning over the RDF models.

#### Institutional Autonomy

##### Requirement

The system shall not store data centrally.

#### Design

ARTEMIS changes the centralized integration paradigm used in antimicrobial resistance surveillance. Unlike other systems[9-11], its distributed architecture does not require centralization of microbiology test results. At query time, a global aggregated view on the local end points is created by the semantic mediator, solving the problem of interoperability while avoiding a central repository, which would violates the project’s legal requirements. Additionally, since there is no need to move data across the health care border, this design gives full control to participating sites, allowing them to stop sharing data at any moment. Further, no historical information for the respective site is kept on the system.

#### Example

In the model–view–control pattern [37] presented in Figure 2, persistent data stores are deployed only within the demilitarized zone of the data providers. The central mediator process and aggregates query constraints locally. In this configuration, there is no need to move datasets with information at the patient level out of the institutional borders. Only aggregated population data are retrieved at query time. Furthermore, institutions can stop sharing data at any moment by shutting down the lCDR server. This change is automatically reflected in ARTEMIS, which will not be able to retrieve any data from the respective data source; other sources remain seamlessly reachable.

#### Knowledge Representation

##### Requirement

The system shall implement a formal and semantic-aware data model.

##### Design

**Figure 5:**
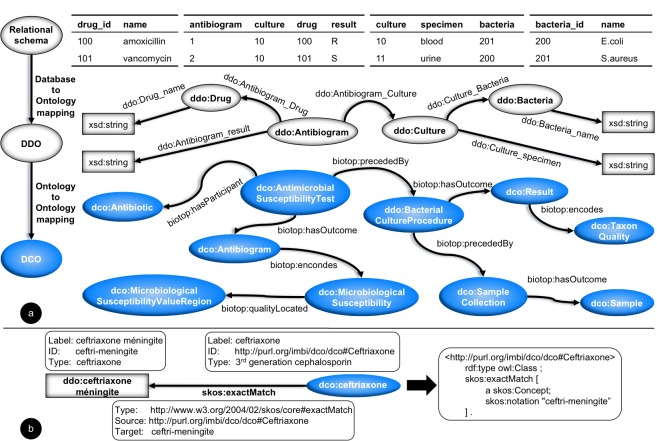
The hybrid ontology-driven interoperability mapping model. White elements represent local-level concepts and blue elements represent shared knowledge. (a) Local entity-relationship schemata are formalized by the data definition ontologies (DDOs). Mappings between DDO data elements and DebugIT Core Ontology (DCO) link local concepts to the global knowledge. (b) Example of a semantic mapping: concept map diagram (left) and RDF/Notation3 representation (right).

In a multinational environment, the contents of electronic health records and laboratory information systems are expressed in several languages and different terminologies. Additionally, spelling mistakes and abbreviations are common in concept definitions. These ambiguities reduce the quality of the statistical analysis. To have unified semantics across the different data sources, in ARTEMIS’s knowledge model (Figure 5), concepts are represented using a formal language (RDF/OWL). Further, they are aligned into common syntaxes defined by biomedical terminologies. Finally, to have a common meaning across the whole system, these formally represented terminologies are mapped to a shared domain ontology.

##### Example

In ARTEMIS, standard terminologies such as the Systematized Nomenclature of Medicine-Clinical Terms (SNOMED-CT), the WHO’s Anatomical Therapeutic Chemical (WHO-ATC) classification system, and the Universal Protein Resource (UniProt/NEWT) are mapped to DebugIT Core Ontology using the Simple Knowledge Organization System (SKOS) ontology [45] and Notation3 rules (Figure 5b). If local concepts are not already defined using these terminologies, they are normalized against them using automatic classification tools [43,44]. Alternatively, local concepts represented in the SKOS notation can be directly mapped to DebugIT Core Ontology.

### Performance Requirement

We assessed the mediator’s SPARQL query performance for query templates 1, 2, and 3. A query mix composed of 225 unique queries, spanning 4 years in daily, monthly, and yearly periods, were used. Each query mix was submitted 10 times against the seven end points. Table 2 summarizes the results. The mean query response time was 4.3 (SD 0.1×10^2^) seconds. Comparing the results with a different aggregation strategy, based on central reasoning, the average retrieval time increased almost 30-fold (mean 130.5, SD 0.1×10^3 ^seconds).

**Figure 6:**
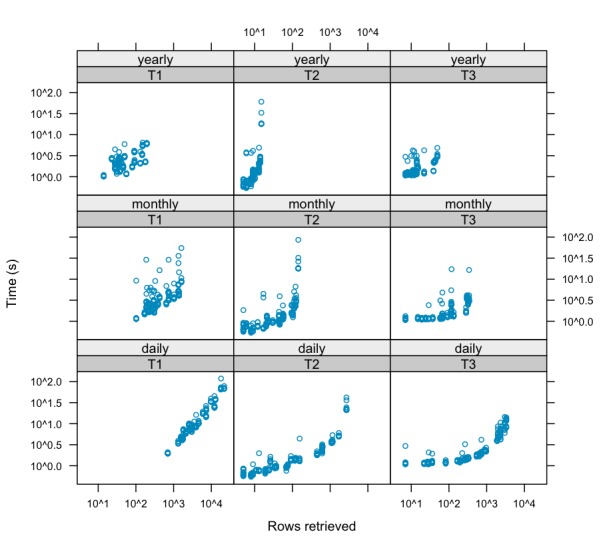
Query performance. Response time and rows retrieved by template (1-3) and aggregation period. As the number of rows retrieved increases, the response time tends also to increase.

Figure 6 shows how the response time of ARTEMIS queries varied with the number of rows retrieved for different query templates and aggregation periods. Indeed, the response time is highly correlated with the number of rows retrieved (ρ = .81, *P *< .001).

**Table 2:** Arithmetic (t_a_) and geometric (t_g_) mean (SD) execution times for the two query mediation strategies: local (Antimicrobial Resistance Trend Monitoring System [ARTEMIS]) versus central (baseline) reasoning.

Template	Number of distinct queries	ARTEMIS		Baseline	
		t_a_ (seconds)	t_g_ (seconds)	t_a_ (seconds)	t_g_ (seconds)
1	75	8.4 (0.1×10^2^)	4.2 (0.1)	311.0 (0.9×10^3^)	308.3 (0.1)
2	75	2.3 (0.6×10)	1.3 (0.1)	74.7 (0.6×10^2^)	72.1 (0.1)
3	75	2.0 (0.2×10)	1.7 (0.1)	5.9 (0.8×10)	2.7 (0.1)
All	225	4.3 (0.1×10^2^)	2.1 (0.1)	130.5 (0.1×10^3^)	39.2 (0.1)

### Result Reliability Requirement

Following the data selection criterion, we created 221 queries for EARS-Net and 153 for SEARCH based on template 1. Table 3 shows the geometric mean resistance rates extracted from the three systems. The results yielded a strong positive correlation coefficient between ARTEMIS and both EARS-Net (ρ = .86, *P *< .001) and SEARCH (ρ = .84, *P *< .001) reference systems.

**Figure 7:**
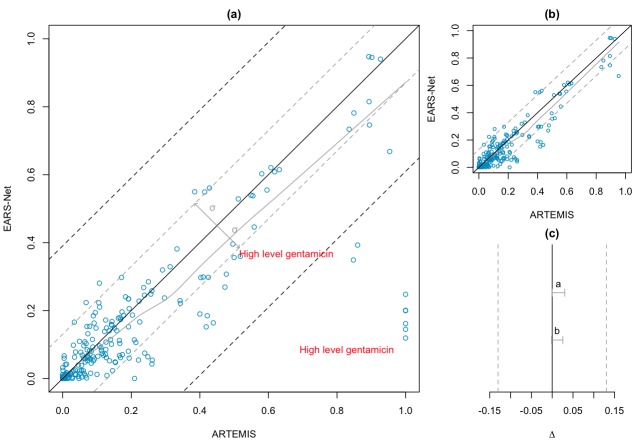
Antimicrobial Resistance Trend Monitoring System (ARTEMIS) vs European Antimicrobial Resistance Surveillance Network (EARS-Net). (a) Resistance rates (n = 221). Black line indicates an exact match (100% equivalence). Gray line indicates best fit. Gray dashed lines indicate Δ = ±0.130. (b) Resistance rates without outliers (n = 213). (c) Gray vertical dashed lines indicate similarity region Δ. Gray horizontal bars indicate two one-sided convolution confidence interval (CI). 95% CI^a^ 0–0.030 (*P* < .001); 95% CI^b^ 0.002–0.026 (*P* < .001).

**Figure 8:**
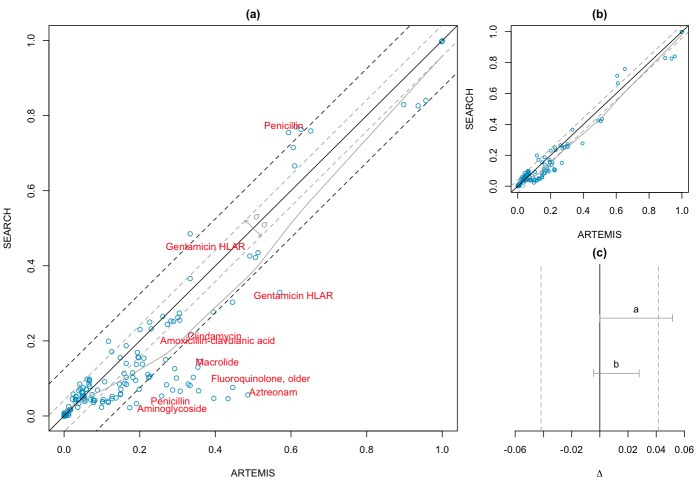
Antimicrobial Resistance Trend Monitoring System (ARTEMIS) vs Sentinel Surveillance of Antibiotic Resistance in Switzerland (SEARCH). (a) Resistance rates (n = 153). Black line indicates exact match (100% equivalence). Gray line indicates best fit. Gray dashed lines indicate Δ = ±0.042. (b) Resistance rates without outliers (n = 143). (c) Gray vertical dashed lines indicate similarity region Δ. Gray horizontal bars indicate two one-sided convolution confidence interval (CI). 95% CI^a^ 0–0.052 (*P* = .17); 95% CI^b^ –0.004 to 0.028 (*P* = .004).

The within-country geometric standard deviation of EARS-Net was σ_ears_ = 0.130. This value was extrapolated to the similarity region Δ ( Δ = σ_ears_) of the two one-sided convolution test. Figure 7 (part a, all results and part b, without outliers) presents the correlation between the two systems, and Figure 7c shows the regions of similarity. The confidence interval (CI) lies in the region of similarity (95% CI 0–0.030; *P *< .001), confirming the equivalence between the ARTEMIS and EARS-Net resistance rates. Similarly, for SEARCH, the Swiss region’s geometric standard deviation was σ_search_ = 0.042, indicating a small susceptibility rate variation in the different regions. In this scenario, the results of ARTEMIS (Figure 8, part a) cannot be considered equivalent to SEARCH (95% CI 0–0.052; *P *= .18). However, removing outliers—that is, those results that fall within a difference in resistance rate bigger than 3σ_search_ (Figure 8, part b)—also leads to an equivalent outcome (95% CI –0.004 to 0.028; *P *= .004).

**Table 3:** Resistance rate geometric mean (SD) and correlation results.

Number of queries	Resistance rate			ρ	*P *value
	EARS-Net^a^	SEARCH^b^	ARTEMIS^c^		
221	0.032 (0.002×10^2^)	NA^d^	0.038 (0.002×10^2^)	.86	<.001
153	NA	0.042 (0.001×10^2^)	0.053 (0.002×10^2^)	.84	<.001

^a ^European Antimicrobial Resistance Surveillance Network.

^b ^Sentinel Surveillance of Antibiotic Resistance in Switzerland.

^c ^Antimicrobial Resistance Trend Monitoring System.

^d ^Not applicable.

## Discussion

In this paper, we present an online and source-independent architecture that enables monitoring of multinational microbiology databases. The system was implemented and deployed in a pilot surveillance network distributed across Europe. From the results, one can see that Semantic Web-based architectures such as that of ARTEMIS are suitable to automate the integration and interoperability of distributed microbiology laboratory data sources. Therefore, it can be used to enable automatic access to antimicrobial resistance information in a transnational context and foster real-time multinational biosurveillance. The architecture is able to interoperate heterogeneous networks via the use of semantic maps that account for local specificity. The data integration process is performed on the fly using standard end points powered with RDF/SPARQL communication, which are mediated by a central engine. The local end points are directly connected to the laboratories’ databases and as such are able to provide (near) real-time resistance information, while avoiding centralization of the data.

### System Architecture

The data integration architecture proposed in ARTEMIS is distinct from existing antimicrobial resistance surveillance systems [14,17,18], as it implements a loosely coupled data federation design[46], which is realized by formalization of the data sources and data semantics. Thus, the data layer is detached from the central system, which avoids central storage and guarantees to care providers full control over the local information. Moreover, online semantic data repositories automate access to local antimicrobial resistance databases, allowing the system to retrieve near real-time antimicrobial resistance trends. Therefore, emerging and outbreak resistances can be easily monitored on a multinational scale. Finally, instead of predetermined and statically monitored bacteria–antibiotic pairs, the architecture introduced here facilitates the expansion of the concept coverage, making the process of tracking resistance of new antibiotics and bacteria trivial. Since concepts are fully formalized by ontologies through the whole architecture, to add a new item to be monitored it is only necessary to create the respective class in the domain ontology and represent it in the semantic mappings (global, local, or both). Thus, it will be automatically reflected in the user interface, including past occurrences of the given class in microbiology tests.

ARTEMIS uses open Semantic Web technologies to provide technical and semantic interoperability. Semantic data sources create a common technical layer over the local microbiology databases, which can be accessed through a standard query protocol (SPARQL). Since local end points are fully formalized and accessible through the Web, they can be linked to external Web resources, such as the Linked Life Data[47], or reused in other clinical research projects to leverage knowledge on infectious diseases by combining different sources of information. Another benefit of using ontologies to represent data is the hierarchical structure, which allows higher-level representation of concepts. Therefore, the system can handle complex queries expressed at group levels allowing, for example, automatic clustering of antibiotic classes such third-generation cephalosporin or bacteria families such as Enterobacteriaceae.

Finally, the powerful query interface allied with the availability of near real-time results makes ARTEMIS not only useful to bodies concerned with supranational resistance but also potentially beneficial to local needs, especially if connected to online prescribing systems for empirical treatments. In addition, this local application might facilitate the maintenance of the system by health care institutions. As Goble and Stevens discussed [46], data integration systems tend to become “data mortuaries” once the research funding ends. Local appeal can possibly help to change this pattern.

### Performance

All SPARQL performance benchmarks presented in the literature are focused on local single-source servers[48]. Thus, they are not adequate to assess the performance of data integration systems. Hence, the ARTEMIS semantic mediator was compared with a standard approach of retrieving and aggregating centrally. As Table 2 shows, the push-down procedure has reduced the retrieval time by 30-fold (19-fold considering the geometric mean). Indeed, as Figure 6 shows, in a distributed system, response time is nearly linearly correlated (ρ = .81, *P *< .001) with the amount of data retrieved. Thus, local reasoning is crucial for systems that require fast response time.

The preference for an SQL-to-RDF engine [42] instead of a native RDF triple store to formalize local data sources was due to scalability issues. As Schmidt et al [49] noted, native RDF triple stores can hardly be scaled to answer queries when their size is bigger than a few million triples. At the mediation level, the use of a push-down approach while performing aggregation has proved efficient. The average query response was in the order of a few seconds (mean 4.3, SD 0.1×10^2 ^seconds), which could contribute to the adoption of the system by clinicians, who consider a good response time an important requirement in the system design [50].

### Comparison with Existing Systems

Existing surveillance systems normally use semiautomatic methods to extract antimicrobial resistance rates[51]. Validation and cleansing steps are taken by experts before statistical analysis. In ARTEMIS, this process is fully automated and, as such, errors can be introduced. To validate ARTEMIS resistance trends, we compared antimicrobial resistance rates with European and national reference systems. The results indicated a strong positive correlation between the susceptibility test outcomes. We carried out a second evaluation based on equivalence tests to confirm the trustworthiness of the results. The tests showed that at the limit of 3σ ARTEMIS trends are deemed equivalent to both EARS-Net and SEARCH.

A difference in concept definition between ARTEMIS and the reference systems negatively affected the results. The majority of outliers (18 out of 33) presented in Figure 7a and Figure 8a were caused by semantic ambiguities between concepts. For example, in ARTEMIS, antibiotic definition follows the WHO-ATC classification system terminology, which does not define a single antibiotic concept for penicillin but rather classes including several antibiotics based on penicillin. In SEARCH, this concept is defined as an antibiotic agent. Analogously, the gentamicin definition, which is not related to concentration in ARTEMIS, is defined as *Gentamicin HLAR *in SEARCH and *High level gentamicin *in EARS-Net. These issues were not accentuated in the comparison with EARS-Net because, as expected, the region of similarity was wider than that of SEARCH, which considers only within-country variations. Adoption of standard and formalized terminologies in the eHealth care field and a more dynamic evolution of terminological resources so that they can cover operational needs are part of the semantic solution.

Finally, in statistical analysis, care should be taken with duplicate tests. If all apparent duplicates are ignored indiscriminately, information may be omitted, such as nosocomial infection, whereas inclusion of all tests may skew the results, usually toward augmentation of resistance [10]. In the reference systems, duplicate tests are manually removed. In ARTEMIS, biases were automatically minimized by considering only the unique tests within an episode of care.

### Limitations

In an ontology-based integration system, automatic mapping from global to local ontologies using first-order logic reasoners creates logical inconsistencies because knowledge from the various local ontologies cannot be completely reconciled in the global model[52]. For example, if at site 1 vancomycin-resistant *Enterococcus *is prevalent, this fact is not necessary true for all other sites. A solution, as implemented in ARTEMIS, is to create query templates over the local ontologies. However, as the system expands to a large number of clinical providers, this approach may prove difficult to maintain, since query templates must be defined centrally for each new data source. Nevertheless, this limitation could be easily overcome if local sources provided a datamart with a common data model as proposed in Figure 4a.

Aligning multinational microbiology laboratory results presents several issues. For example, it has been shown[16] that, for a given sample test, independent laboratories will present different outcomes. Differences in susceptibility breakpoint across countries is also a complex issue involving standardization of antibiogram methodologies. Additionally, results of second-line antibiotics tend to present bias toward resistance, since they are normally tested when isolates show resistance to first-line drugs[10]. The methodology proposed here cannot solve most of the intrinsic divergence between different laboratory procedures. Regardless, ARTEMIS does not aim to tackle these issues but rather to promote access to distributed antimicrobial resistance information as soon as data are available in a formalized and semantically defined way.

### Conclusions

We designed, implemented, and deployed the ARTEMIS architecture in a small-scale biosurveillance network of European hospitals. Results indicate that the distributed monitoring architecture introduced here can potentially be used to build transnational antimicrobial resistance surveillance networks. The architecture proved to be efficient and reliable, while complying with local legal and regulatory frameworks. The Semantic Web-based approach proved to be an effective solution for development of eHealth architectures that enable online antimicrobial resistance monitoring from heterogeneous data sources. In the future, we plan to investigate local model mediation, paving the way to a more easily maintainable system. We expect that new health care institutions can join the network so that it can provide clinicians and decision makers with a missing tool to tackle the growing threat of rising emergent infectious diseases and antibiotic resistance patterns.

## Multimedia Appendix 1

Architecture design.

## Multimedia Appendix 2

Equivalence test.

## Abbreviations

ARTEMIS: Antimicrobial Resistance Trend Monitoring System
CI: confidence interval
DebugIT: Detecting and Eliminating Bacteria Using Information Technology
EARS-Net: European Antimicrobial Resistance Surveillance Network
lCDR: local clinical data repository
RDF: Resource Description Framework
SEARCH: Sentinel Surveillance of Antibiotic Resistance in Switzerland
SKOS: Simple Knowledge Organization System
SNOMED-CT: Systematized Nomenclature of Medicine-Clinical Terms
SPARQL: Simple Protocol and RDF Query Language
WHO: World Health Organization
WHO-ATC: World Health Organization- Anatomical Therapeutic Chemical
