# Supplementary material for: Building a Transnational Biosurveillance Network Using Semantic Web Technologies: Requirements, Design, and Preliminary Evaluation
Source: J Med Internet Res. 2012 May 29;14(3):e73. doi: 10.2196/jmir.2043 (PMC3799609; doi:10.2196/jmir.2043)
Supplement: Supplementary file 1 [file jmir_v14i3e73_app1.pdf]

Multimedia Appendix 1: Architecture Design

Information Model

Domain Ontologies

ARTEMIS domain knowledge is formalized by a core ontology, the DebugIT Core Ontology (DCO). DCO uses the OWL language to represent classes and properties. Currently, it contains 1355 classes that cover the complete scope of the project. The main clinical areas described by DCO are microbiology laboratories, diagnoses and medication actions. In order to facilitate the interaction of the end-user with the domain ontology, a subset of DCO is used in the ARTEMIS interface. It omits classes that are not relevant to the AMR queries.

Data Definition Ontologies

Local ontologies are used to describe each semantic endpoint in a formal and computer-understandable language. Each data source's legacy system is described by a data definition ontology (DDO). A DDO is a representation of the microbiology database using the RDF formalism. As shown in Figure 5-a, database tables become RDF classes and columns become RDF properties in the DDO definition. DDOs are exposed in the Web so that local concepts can be linked to the domain knowledge and account for any local specificity.

Mapping Ontologies

To align biomedical terminologies and locally defined concepts coming from the legacy system of the participant sites with the domain ontology, semantic mappings were created using the SKOS ontology (Figure 5-b). At the global level, mappings from SNOMED-CT, WHO-ATC and UniProt terminologies were designed. In case of using other biomedical terminologies or even local terminologies, local mappings need to be provided by each site. This step is important, as it can easily be adapted to support local needs and evolutions.

Run-Time Component Model

Data Model Layer

In the architecture's data layer, local microbiology laboratory databases are converted into semantic endpoints. This is achieved through a fully semantic-complying clinical data repository, the local Clinical Data Repository (lCDR), as further described in [34,35]. The lCDRs are set-up within the de-militarized zone of each site participant in the DebugIT network. The lCDR, as well as other DebugIT services, expose data in the RDF format and communicate using the SPARQL protocol. It is the interface of the DebugIT services to the data providers.

Controller Layer

The semantic mediation process is performed in the controller layer. The query mediator defines, for each lCDR, SPARQL representations of a limited set of AMR clinical questions presented in the view layer. The clinical question SPARQL queries are built as templates, which are parameterized queries using DCO concepts. Assigning values to a clinical question template results in a new SPARQL query. For

example, the template *"What is the antimicrobial resistance evolution to* :antibiotic *of* :pathogen *cultured from* :sample_origin *from* :begin_date *to* :end_date*?"* might be instantiated as *"What is the antimicrobial resistance evolution to* cefepime *of* Escherichia coli *cultured from* blood sample *from* 2011-01-01 *to* 2011-12-31*?"*. Thus, a template represents an infinite number of queries.

At the query run-time, templates expressed through global concepts are translated into local SPARQL queries with terms from the local ontologies. The query parameters are expanded employing the hierarchical information modeled in the domain ontology and are translated to local terms using the semantic mappings. For example, the DCO concept *"3rd generation cephalosporin"* shown in Figure 9 is expanded to its DCO subclasses, which are further mapped to local DDO terms. In order to optimize network performance and reinforce patient confidentiality, aggregation operations are pushed down to the lCDRs. The SPARQL operators *COUNT* and *GROUP BY* are used to perform local result aggregation. Results are fetched respecting the query filter constraints, which perform logical disjunction operations for the expanded parameters. An inverse process is performed on the results retrieved: local terms are translated to global terms, which are aggregated in the root concept, i.e.,*"3rd generation cephalosporin"* in the example.

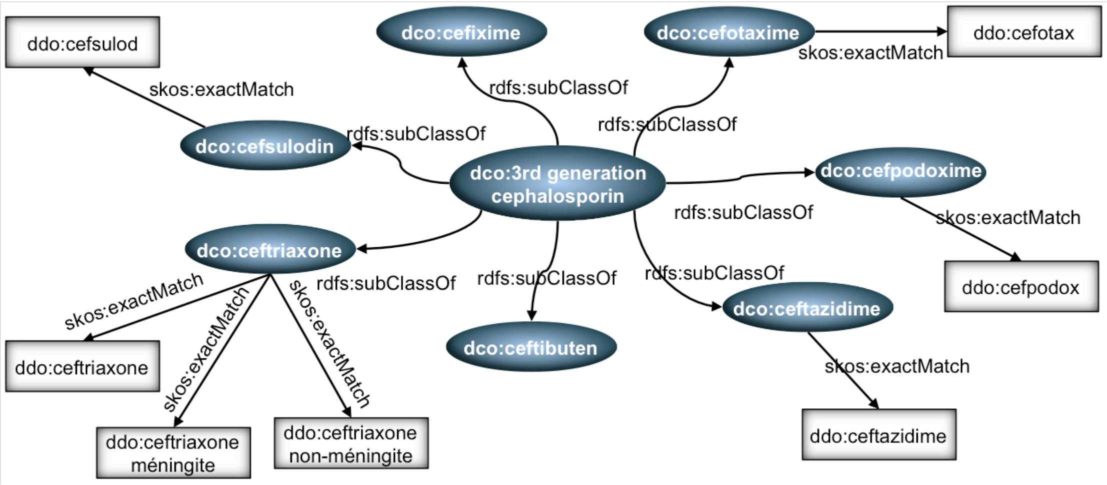

Figure 9: Global-to-local concept translation and query expansion model. Ontology properties, such as *subClassOf*, and the SKOS semantic mapping *exactMatch* are used for query expansion and translation.

View Layer

Finally, the view layer provides methods for users to interact with the system. It implements two main modules: querying input and data visualization. The querying input interface presents a set of clinical question templates and the Interface Ontology input menu, which is used to fill in the template parameters. To improve usability and user-friendliness, query templates are expressed in natural language as in the template *"What is the prevalence of* :antibiotic :susceptibility :pathogen *in* :sample *extracted from* :gender *patients at* :clinical_setting *during period* :begin_date - :end_date*?"*. The visualization module provides functions to extract trends, cumulative sum and other statistics from the data retrieved. Ultimately, it implements a set of charts in order to cover comprehensively the interpretation of the data.

34. Teodoro D, Choquet R, Pasche E, Gobeill J, Daniel C, Ruch P, Lovis C. Biomedical data management: a proposal framework. Stud Health Technol Inform. 2009;150:175-9. [PMID: 19745292]
35. Teodoro D, Choquet R, Schober D, Mels G, Pasche E, Ruch P, Lovis C. Interoperability driven integration of biomedical data sources. Stud Health Technol Inform. 2011;169:185-9. [PMID: 21893739]
