# Supplementary material for: Building a Transnational Biosurveillance Network Using Semantic Web Technologies: Requirements, Design, and Preliminary Evaluation
Source: J Med Internet Res. 2012 May 29;14(3):e73. doi: 10.2196/jmir.2043 (PMC3799609; doi:10.2196/jmir.2043)
Supplement: Supplementary file 2 [file jmir_v14i3e73_app2.pdf]

Multimedia Appendix 2: Equivalence Test

Two one-sided t-test is an equivalence test widely applied in bioequivalence studies [53] but also in model comparison [54]. We employed its variation for non-normal distribution, the two one-sided convolution test (TOSC), in the susceptibility equivalence test. TOSC was based on a Wilcoxon test to derive the confidence interval (CI) [55]. The null hypothesis was that resistance rates of ARTEMIS and of the reference surveillance system differed by at least an interval Δ. ARTEMIS results were deemed equivalent to the reference trends at the $\alpha=0.05$ level if the CI for the difference in resistance rates was completely contained within a region of similarity, delimited by the endpoints $-\Delta$ and $+\Delta$. We used the susceptibility results' standard deviation of different countries in EARS-Net to estimate the region of similarity. Since ARTEMIS rates came from different data samples, we extrapolated this interval as a level of acceptance of the results. We applied a similar procedure for SEARCH but instead of different countries, the standard deviation among the different regions of Switzerland (East, Mid and West) was used.

53. Pigeot I, Hauschke D, Shao J. The bootstrap in bioequivalence studies. J Biopharm Stat. 2011 Nov;21(6):1126-39. [PMID: 22023681]
54. Cribbie RA, Gruman JA, Arpin-Cribbie CA. Recommendations for applying tests of equivalence. J Clin Psychol. 2004 Jan;60(1):1-10. [PMID: 14692005]
55. Hothorn LA, Hasler M. Proof of hazard and proof of safety in toxicological studies using simultaneous confidence intervals for differences and ratios to control. J Biopharm Stat 2008;18(5):915-33. [PMID: 18781525]
